# Risk factors for falls in patients treated with chemotherapy

**DOI:** 10.1186/s40780-025-00502-w

**Published:** 2025-10-27

**Authors:** Shinji Oda, Kenshi Takechi, Chiyuki Tsukui, Satoru Hirai, Shingo Takatori, Takashi Otsuka

**Affiliations:** 1 Department of Pharmacy, Matsuyama Shimin Hospital, Matsuyama, Japan; 2 Department of Drug Information Analysis, College of Pharmaceutical Sciences, Matsuyama University, 4-2 Bunkyo-cho, Matsuyama, Ehime 790-8578 Japan; 3 Department of Rehabilitation, Matsuyama Shimin Hospital, Matsuyama, Japan

**Keywords:** Chemotherapy, Fall, Peripheral neuropathy, Hypoglycemic drugs, Body mass index

## Abstract

**Background:**

Falls are a serious concern for hospitalized patients, as they can lead to a decline in quality of life (QOL) and increased nursing care needs. Chemotherapy-induced peripheral neuropathy (CIPN) increases the risk of falls; however, only a few reports have investigated CIPN in conjunction with other factors. This study aimed to identify risk factors for falls in hospitalized patients undergoing chemotherapy.

**Methods:**

We retrospectively analyzed 21,717 hospitalized patients, including 443 who received chemotherapy, at Matsuyama Shimin Hospital between April 2016 and March 2023. Multivariate logistic regression analysis was performed to assess the fall risk in hospitalized patients who received chemotherapy.

**Results:**

Among 21,717 hospitalized patients, 930 (4.3%) experienced at least one fall. Multivariate logistic regression identified 13 factors, including age, sex, BMI, and mobility assistance. Notably, chemotherapy showed the highest odds ratio among these factors (OR 3.40, 95% CI 2.49–4.65). In the chemotherapy subgroup (*n* = 443), multivariate analysis identified five factors (body mass index (BMI); decline in judgment, comprehension, and memory; treatment with hypoglycemic drugs; treatment with high-risk CIPN drugs; and lung cancer). The fall rate was significantly higher in patients who received both hypoglycemic drugs and high-risk CIPN drugs (37.5%, 6/16) than in those who received either factor alone (14.1%, 27/192; *p* < 0.05).

**Conclusions:**

Chemotherapy was identified as an independent risk factor for falls. Among patients receiving chemotherapy, both hypoglycemic drugs and high-risk CIPN drugs were associated with an increased risk of falls, and the fall rate was significantly higher in those treated with both drugs. Therefore, these patients should be carefully monitored for fall risk.

## Background

Falls among hospitalized patients are considered an important safety problem because they can lead to a decline in quality of life (QOL) and increased nursing care needs [1]. These falls result from a complex interplay of multiple risk factors, including age, history of falls, assistance with activities of daily living (ADLs), use of psychotropic or hypnotic medications, and cognitive dysfunction [2–4]. Results of a multivariate logistic regression analysis in a previous study revealed that older inpatients with malignant tumors had the highest risk of falling [5, 6]. However, patients with malignant tumors, regardless of age, may have an increased risk of falls due to muscle weakness, impaired balance associated with the malignant tumors, and treatments such as chemotherapy and surgery [7]. Therefore, fall-prevention in patients with malignant tumors, regardless of age, is critically important.

Chemotherapy-induced peripheral neuropathy (CIPN) causes sensory neuropathy, motor, and autonomic neuropathy, affecting daily functioning and social participation [8–10]. Furthermore, worsening of CIPN increases the risks of tripping and falling [11]. Among the symptoms of CIPN, motor neuropathy increases the risk of falls owing to worsened gait and balance caused by muscle atrophy, muscle weakness, flaccidity, and paralysis [12]. Notably, CIPN is more likely a result of treatment with platinum compounds, taxanes, and vinca alkaloids [13]. Although these high-risk CIPN drugs are widely used in Japan, detailed studies examining their relationship with other fall risk factors are lacking. The identification of risk factors for falls in patients undergoing chemotherapy could contribute to reducing the number of hospitalized patients who experience falls. Therefore, in this study, we aimed to analyze the risk factors of falls in patients who underwent chemotherapy.

## Methods

### Study period and patients

**Fig. 1:**
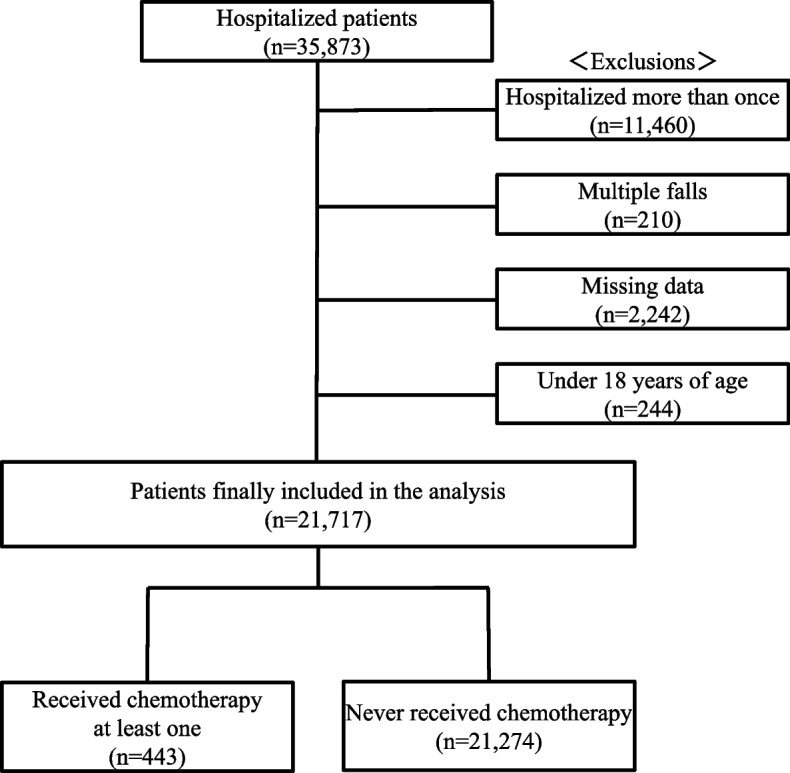
Scheme depicting steps involved in sample selection

This study included all patients who were hospitalized at Matsuyama Shimin Hospital between April 2016 and March 2023 (35,873 patients). The following exclusion criteria were applied with reference to our previous study [5]: multiple hospitalizations during the study period (11,460 patients), multiple falls during the study period (210 patients), incomplete data (2,242 patients), and age < 18 years (244 patients). After applying the exclusion criteria, 21,717 patients were finally included. In addition, patients were divided into those who had received chemotherapy at least once (*n* = 443) and those who had never received chemotherapy (*n* = 21,274) (Fig. 1).

### Patient characteristics

We selected variables primarily based on data available in electronic medical records and nursing assessments, and we also consulted our previous study, which used a similar approach to identify factors associated with falls in hospitalized patients [5]. Data extracted from electronic medical records included age, sex, body mass index (BMI), assessment of fall risk by nurses at admission, prescribed drugs (excluding topical treatments but including as-needed drugs), history of chemotherapy, exposure to high-risk CIPN drugs (oxaliplatin, paclitaxel, nab-paclitaxel, or vincristine, which are associated with high peripheral neuropathy rates in clinical trials. cisplatin, docetaxel, bortezomib and thalidomide were excluded due to their low prescription frequency), and cancer type for patients who underwent chemotherapy. For patients who experienced a fall, the most recent data recorded prior to the fall event were used for analysis. In this study, fall was defined as “an unintentional grounding event on the ground, floor, or a lower surface” [14].

### Fall risk assessments

Fall risk assessment by nurses was conducted according to standardized evaluations validated in on our previous study [5]. The definitions of each assessment by nurses are detailed below.

- Mobility assistance: unable to transfer independently, requiring assistance from others.
- Dysfunction of the lower extremities: paralysis, numbness, edema, contracture, deformity, and amputation of the lower extremities.
- Muscle weakness: inability to perform any of the following five tests: lower limb extension and raising, knee-stand glute raises, ankle dorsiflexion, seated hip flexion, or grasping a towel.
- Visual disturbance: diplopia, visual field constriction, hemianopia, and complete blindness.
- Hearing impairment: hearing aid users or patients who cannot hear unless spoken to directly into the ear.
- Bed rest: doctor-ordered restrictions.
- Decline in judgment, comprehension, and memory: patients who have any of the following issues: inability to hold a coherent conversation, difficulty communicating, inconsistent or illogical narratives, frequent repetition, inability to answer questions, or failure to understand explanations.

### Statistical analysis

The Fisher’s exact test or Student’s t-test was used to perform between-group comparisons of the presence or absence of falls. For the analysis of risk factors for falls, odds ratios were calculated using multivariate logistic regression analysis. The covariate factors selected in this study reflected the clinical symptoms and were considered related to falls. After confirming a correlation coefficient < 0.4, variance inflation factor < 10, and the absence of collinearity for each factor, statistical analysis was performed using EZR version 1.54, with the significance level set at < 5% [15].

### Ethical considerations

This study was conducted in accordance with the ethical guidelines for medical research involving human subjects and the Declaration of Helsinki. It was approved by the ethics committee of the hospital (approval number: 20190731mshe). Owing to the retrospective study design, consent was obtained from each patient using an opt-out document posted on the website of the hospital.

## Results

### Patient characteristics

As shown in Table 1, a total of 930 patients (4.2%) experienced falls during the study period. In univariate analyses, 11 of the 16 variables, including age, BMI, and mobility assistance, were significantly associated with falls. Multivariate logistic regression identified 13 independent risk factors, such as age, sex, BMI, and mobility assistance. Among these, chemotherapy showed the highest odds ratio (OR 3.40, 95% CI 2.49–4.65, *p* < 0.001).

**Table 1:** Patient characteristics

Factor	No fall	Fall	Univariate		Multivariate	
			Odds ratio (95% CI)	*P* value	Odds ratio (95% CI)	*P* value
N	20,787	930				
Age, years [mean(SD)]	68.9 (18.6)	78.8 (11.9)	1.04 (1.04–1.05)	< 0.001	1.03 (1.02–1.04)	< 0.001
Male sex, n(%)	10,708 (51.5)	502 (54.0)	1.10 (0.97–1.26)	0.149	1.37 (1.19–1.57)	< 0.001
BMI, kg/m2 [mean(SD)]	22.6 (4.24)	21.4 (4.06)	0.93 (0.92–0.95)	< 0.001	0.97 (0.95–0.99)	< 0.001
Mobility assistance, n(%)	6,984 (33.6)	545 (58.6)	2.80 (2.45–3.20)	< 0.001	1.52 (1.29–1.79)	< 0.001
Toilet use at night, n(%)	9,152 (44.0)	432 (46.5)	1.10 (0.97–1.26)	0.147	0.94 (0.82–1.09)	0.41
Dysfunction of the lower extremities, n(%)	2,522 (12.1)	183 (19.7)	1.77 (1.50–2.10)	< 0.001	1.25 (1.04–1.49)	0.014
Muscle weakness, n(%)	8,429 (40.5)	618 (66.5)	2.90 (2.53–3.34)	< 0.001	1.46 (1.23–1.73)	< 0.001
Visual disturbance, n(%)	1,559 (7.5)	95 (10.2)	1.40 (1.13–1.75)	0.004	1.26 (1.00–1.58)	0.047
Hearing impairment, n(%)	1,140 (5.5)	63 (6.8)	1.25 (0.96–1.63)	0.092	0.66 (0.50–0.86)	0.0026
Bed rest, n(%)	2,351 (11.3)	124 (13.3)	1.21 (0.99–1.46)	0.065	0.76 (0.62–0.94)	0.01
Decline in judgment, comprehension, and memory, n(%)	2,887 (13.9)	296 (31.8)	2.89 (2.51–3.34)	< 0.001	1.44 (1.22–1.71)	< 0.001
Analgesic, n(%)	875 (4.2)	50 (5.4)	1.29 (0.97–1.73)	0.096	1.43 (1.06–1.93)	0.021
Hypoglycemic drugs, n(%)	2,007 (9.7)	111 (11.9)	1.27 (1.03–1.55)	0.024	1.22 (0.99–1.51)	0.065
Antihypertensive drugs and diuretics, n(%)	5,647 (27.2)	305 (32.8)	1.31 (1.14–1.51)	< 0.001	0.95 (0.82–1.10)	0.49
Hypnotics, n(%)	5,751 (27.7)	444 (47.7)	2.39 (2.09–2.73)	< 0.001	2.06 (1.80–2.35)	< 0.001
Chemotherapy, n(%)	392 (1.9)	51 (5.5)	3.02 (2.24–4.07)	< 0.001	3.40 (2.49–4.65)	< 0.001

*CI* confidence interval, *BMI* body mass index

### Patient characteristics who underwent chemotherapy

Patient characteristics of those who underwent chemotherapy are listed in Table 2. In total, 51 (11.5%) out of 443 patients treated with chemotherapy experienced falls. Univariate analysis of each item for fall status showed significant differences in six items (BMI; mobility assistance; decline in judgment, comprehension, and memory; treatment with hypoglycemic drugs; treatment with high-risk CIPN drugs; and lung cancer). Multivariate logistic regression analysis (Case 1) was then conducted, including all variables that were significant in univariate analysis, along with age and sex (Case 1). This analysis identified five independent risk factors for falls: lower BMI (OR 0.90, 95% CI 0.82–0.98, *p* = 0.012), decline in judgment, comprehension, and memory (OR 3.77, 95% CI 1.43–9.93, *p* = 0.007), treatment with hypoglycemic drugs (3.47, 95% CI 1.52–7.93, *p* = 0.003), treatment with high-risk CIPN drugs (OR 2.20, 95% CI 1.17–4.12, *p* = 0.014), and lung cancer (OR 2.00, 95% CI 1.00–3.99, *p* = 0.049). To address the potential issue of overfitting, we performed additional sensitivity analyses. In a parsimonious model including five clinically relevant covariates (age, sex, BMI, hypoglycemic drugs, and high-risk CIPN drugs; Case 2) and another minimal model including only hypoglycemic drugs and high-risk CIPN drugs (Case 3), both variables consistently remained significantly associated with falls (Table 3).

**Table 2:** Patient characteristics who underwent chemotherapy

Factor	No fall	Fall
N	392	51
Age, years [mean(SD)]	70.8 (11.5)	72.9 (10.3)
Male sex, n(%)	228 (58.2)	31 (60.8)
BMI, kg/m2 [mean(SD)]	22.0 (4.12)	20.4 (3.96)
Mobility assistance, n(%)	92 (23.5)	22 (43.1)
Toilet use at night, n(%)	279 (71.2)	36 (70.6)
Dysfunction of the lower extremities, n(%)	37 (9.44)	8 (15.7)
Muscle weakness, n(%)	136 (34.7)	25 (49.0)
Visual disturbance, n(%)	32 (8.16)	3 (5.88)
Hearing impairment, n(%)	15 (3.83)	2 (3.92)
Bed rest, n(%)	7 (1.79)	1 (1.96)
Decline in judgment, comprehension, and memory, n(%)	19 (4.85)	9 (17.6)
Analgesic, n(%)	11 (2.81)	3 (5.88)
Hypoglycemic drugs, n(%)	36 (9.18)	11 (21.6)
Antihypertensive drugs and diuretics, n(%)	117 (29.8)	12 (23.5)
Hypnotics, n(%)	130 (33.2)	19 (37.3)
High-risk CIPN drugs, n(%)	149 (38.0)	28 (54.9)
Gastrointestinal cancer, n(%)	210 (53.6)	23 (45.1)
Lung cancer, n(%)	77 (19.6)	18 (35.3)
Hematologic cancer, n(%)	47 (12.0)	4 (7.8)
Breast cancer, n(%)	31 (7.9)	3 (5.9)
Other cancers, n(%)	27 (6.9)	3 (5.9)

*BMI* body mass index, *CIPN* chemotherapy-induced peripheral neuropathy

**Table 3:** Univariate and multivariate logistic regression analyses of risk factors for falls in patients who underwent chemotherapy

Factor	Univariate		Multivariate (Case 1)		Multivariate (Case 2)		Multivariate (Case 3)	
	Odds ratio (95% CI)	*P* value	Odds ratio (95% CI)	*P* value	Odds ratio (95% CI)	*P* value	Odds ratio (95% CI)	*P* value
Age, years	1.02 (0.99–1.05)	0.226	1.01 (0.98–1.04)	0.640	1.02 (0.99–1.05)	0.230		
Male sex	1.11 (0.61–2.03)	0.764	0.94 (0.49–1.78)	0.840	1.09 (0.59–2.02)	0.780		
BMI, kg/m2	0.90 (0.83–0.97)	0.007	0.90 (0.82–0.98)	0.012	0.89 (0.82–0.97)	0.005		
Mobility assistance	2.47 (1.36–4.51)	0.004	1.47 (0.75–2.91)	0.260				
Toilet use at night	0.97 (0.51–1.84)	1.000						
Dysfunction of the lower extremities	1.79 (0.78–4.08)	0.213						
Muscle weakness	1.81 (1.01–3.26)	0.062						
Visual disturbance	0.70 (0.21–2.38)	0.784						
Hearing impairment	1.03 (0.23–4.62)	1.000						
Bed rest	1.10 (0.13–9.13)	1.000						
Decline in judgment, comprehension, and memory	4.21 (1.79–9.89)	0.002	3.77 (1.43–9.93)	0.007				
Analgesic	2.16 (0.58–8.03)	0.211						
Hypoglycemic drugs	2.72 (1.28–5.76)	0.013	3.47 (1.52–7.93)	0.003	3.33 (1.50–7.37)	0.003	2.92 (1.36–6.25)	0.006
Antihypertensive drugs and diuretics	0.72 (0.37–1.43)	0.414						
Hypnotics	1.20 (0.65–2.19)	0.637						
High-risk CIPN drugs	1.99 (1.10–3.57)	0.023	2.20 (1.17–4.12)	0.014	2.00 (1.09–3.68)	0.025	2.09 (1.15–3.79)	0.016
Gastrointestinal cancer	0.71 (0.40–1.28)	0.260						
Lung cancer	2.23 (1.19–4.17)	0.012	2.00 (1.00–3.99)	0.049				
Hematologic cancer	0.63 (0.22–1.81)	0.390						
Breast cancer	0.73 (0.21–2.47)	0.610						
Other cancers	0.85 (0.25–2.89)	0.790						

Multivariate (Case 1): Full model including all candidate variables. Multivariate (Case 2): Parsimonious model including five clinically relevant covariates (age, sex, BMI, hypoglycemic drugs, high-risk CIPN drugs). Multivariate (Case 3): Minimal model including only hypoglycemic drugs and high-risk CIPN drugs

*CI* confidence interval, *BMI* body mass index, *CIPN* chemotherapy-induced peripheral neuropathy

### Fall rates of patients treated with high-risk CIPN drugs and hypoglycemic drugs

**Fig. 2:**
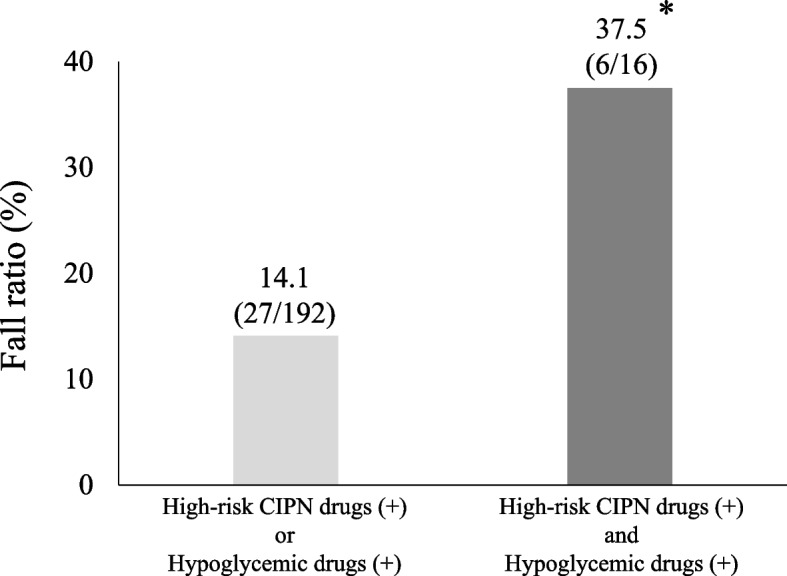
Fall rates of patients treated with high-risk CIPN drugs and hypoglycemic drugs. *p < 0.05 vs high-risk CIPN drugs (+) or hypoglycemic drugs (+). CIPN, chemotherapy-induced peripheral neuropathy

As shown in Fig. 2, patients who received both hypoglycemic drugs and high-risk CIPN drugs had a significantly higher fall rate (37.5%, 6/16) compared with those who received either factor alone (14.1%, 27/192; *p* < 0.05).

## Discussion

In this study, the overall fall rate was 4.2% (930/21274), whereas the fall rate among patients who underwent chemotherapy was 11.5% (51/443), indicating a higher risk of falls due to chemotherapy. Multivariate logistic regression analysis of the overall patient population identified 13 independent risk factors, including age, sex, BMI, and mobility assistance. Among these, chemotherapy showed the highest odds ratio. In the subgroup of patients who received chemotherapy, multivariate analysis revealed that lower BMI; decline in judgment, comprehension, and memory; hypoglycemic drugs; high-risk CIPN drugs; and lung cancer were significantly associated with falls.

BMI and decline in judgment, comprehension, and memory were identified as risk factors in both the overall and chemotherapy-specific analyses, suggesting that they are important risk factors for falls. Particularly with regards to BMI, O’Neil et al. found an association between low BMI and increased risk of hospital falls [16]. The side effects of chemotherapy (e.g., nausea, loss of appetite, and taste disorders) and cancer cachexia can reduce food intake and lead to a decrease in BMI. Therefore, patients with low BMI undergoing chemotherapy may need appropriate nutritional support to prevent falls.

In the chemotherapy subgroup, patients receiving both hypoglycemic drugs and high-risk CIPN drugs exhibited a markedly higher fall rate compared with those receiving either factor alone, suggesting a potential interaction between diabetes and chemotherapy-induced peripheral neuropathy. Previous studies have reported that treatment with high-risk CIPN drugs is associated with increased fall risk [11, 17]. Additionally, a meta-analysis indicated that older adults with diabetes mellitus have an increased risk of falls [18], and diabetic peripheral neuropathy has also been identified as an important risk factor for falls [19]. Analyses of phase II/III trials of taxanes by the Southwest Oncology Group further showed that diabetes is a risk factor for CIPN [20], and a large cohort study of patients with lung cancer demonstrated that diabetes increases the risk of CIPN in patients treated with platinum compounds and taxanes [21]. Taken together, these findings suggest that diabetic patients receiving high-risk CIPN drugs are particularly susceptible to developing CIPN, which in turn increases their fall risk. Clinically, this finding underscores the need for careful monitoring and targeted fall-prevention strategies in patients treated with both hypoglycemic drugs and high-risk CIPN drugs.

In the chemotherapy subgroup analysis, lung cancer was associated with an increased risk of falls, whereas other cancer types were not significant. Previous studies have also reported that lung cancer survivors demonstrate impaired dynamic balance and higher fall frequency compared with healthy controls [22]. However, given the limited number of fall events within each cancer type, this result should be interpreted cautiously as a cohort-specific finding. Further studies are warranted to clarify whether lung cancer itself or its treatment regimens underlie the observed increased risk. Nevertheless, the use of hypnotics, generally regarded as risk factors for falls in hospitalized patients, was not significantly associated with falls in our chemotherapy subgroup. This may be due to the relatively small sample size and heterogeneity of hypnotic drug classes, which could have diluted potential effects.

This study has several limitations. First, it was a retrospective study conducted at a single institution, and there may have been biases regarding patient backgrounds. Second, to isolate the effects of fall history and prescribed medications, we excluded patients with multiple hospitalizations, who may inherently have a higher fall risk and unique contributing factors. Third, we were unable to analyze ADL, performance status, comorbidities (e.g., heart failure, stroke, dementia), environmental factors, patient-specific factors (e.g., social support, hospital room settings), outcomes of falls (e.g., fractures), fall risk scores, and the presence and severity of diabetes. In this study, diabetes was defined based on the use of hypoglycemic drugs, as data on formal diabetes diagnoses were not consistently available. Therefore, the impact of diabetes severity or complications on fall risk could not be directly assessed and should be considered when interpreting the results. Inclusion of these factors could have provided a more comprehensive evaluation of fall risk. Fourth, we did not examine variables such as time from admission to fall occurrence, use of anti-allergic drugs associated with chemotherapy, therapeutic drugs for CIPN, cumulative dosage and treatment duration of high-risk CIPN drugs, time since chemotherapy completion, or the occurrence and severity of CIPN, and biochemical parameters such as glycemic control and chemotherapy-related anemia. These unexamined variables may have acted as confounding factors, particularly regarding the interaction between diabetes and CIPN. Fifth, because this study only included inpatients, the fall status of outpatients treated with high-risk CIPN drugs was not analyzed. Finally, the limited number of fall events within specific cancer types and subgroups may affect the generalizability of our findings. Future studies employing prospective designs, multi-center cohorts, detailed chemotherapy regimens, CIPN severity assessments, and comprehensive patient data are warranted to validate and extend our results.

## Conclusions

Chemotherapy is an independent risk factor for falls among hospitalized patients. In patients receiving chemotherapy, treatment with both hypoglycemic drugs and high-risk CIPN drugs was associated with a substantially increased risk of falls, suggesting a potential interaction between diabetes and chemotherapy-induced peripheral neuropathy. These findings underscore the need for careful monitoring and implementation of targeted fall-prevention strategies in this high-risk population. Future studies incorporating prospective designs, detailed assessment of CIPN severity, chemotherapy regimens, and comprehensive patient factors are warranted to further clarify fall risk and guide clinical interventions.

## Data Availability

All data generated or analyzed during this study are included in this published article.

## Abbreviations

ADL: Activities of daily living
BMI: Body mass index
CIPN: Chemotherapy-induced peripheral neuropathy
PS: Propensity score
QOL: Quality of life
